# Qualitative Evaluation of the Seated Physical Activity INtervention (SPIN) Randomized Controlled Trial for Wheelchair Users with Multiple Sclerosis (MS): Formative Feedback and Future Directions

**DOI:** 10.3390/healthcare14131824

**Published:** 2026-06-23

**Authors:** Angela J. Piasecki, Robert W. Motl, Katherine Froehlich-Grobe, Stephanie L. Silveira

**Affiliations:** 1 Department of Management, Policy, and Community Health, UTHealth Houston School of Public Health, Houston, TX 77030, USA; angelajpiasecki@outlook.com; 2 Department of Kinesiology and Nutrition, University of Illinois Chicago, Chicago, IL 60612, USA; robmotl@uic.edu; 3 Research Department, Craig Neurorehabilitation and Research Hospital, Englewood, CO 80113, USA; kfroehlich-grobe@craighospital.org

**Keywords:** wheelchair, exercise, multiple sclerosis, qualitative

## Abstract

Background/Objectives: Wheelchair users with multiple sclerosis (MS) often face barriers that restrict participation in physical activity and exercise training. This manuscript reports on participant feedback to guide evaluating and refining a novel exercise training program, Seated Physical activity INtervention (SPIN). SPIN was adapted from the Guidelines for Exercise in MS (GEMS) approach using a three-step community-engaged research framework based on meeting the needs of wheelchair users with MS. Methods: Semi-structured interviews were conducted with 9 participants who completed the 16-week SPIN intervention. The key SPIN intervention components were the exercise prescription, exercise equipment, and behavioral coaching grounded in Social Cognitive Theory. Formative interview domains included overall experience, enjoyable and missing components, delivery modifications, barriers, lessons learned, and additional research topics of interest. Data were analyzed and reported using a rapid qualitative analysis approach. Results: Interviews averaged 16 ± 10 min. Participants reported enjoying SPIN, noting program strengths as being flexible and appropriate for individuals with MS, receiving coaching calls by knowledgeable staff that offered support and accountability, and receiving exercise equipment and video demonstrations. Participants also identified strategies for enhancing the program such as including peer support, offering real-time feedback during exercise, and adding other wellness behavior topics (e.g., diet). Conclusions: The results offer helpful ideas to consider when developing exercise training programs for wheelchair users with MS and other disabilities that may improve health and well-being.

## 1. Introduction

Multiple sclerosis (MS) is a neurodegenerative disease of the central nervous system characterized by progressive functional decline. Among individuals with progressive phenotypes, wheelchair use becomes more common, alongside reduced access to high-efficacy disease-modifying therapies [1,2]. Exercise training is a well-established, evidence-based strategy for managing symptoms and slowing disease progression [3,4]. Individuals with MS who use wheelchairs face persistent barriers to engaging in physical activity and exercise training [5,6]. The barriers can include mobility limitations, fatigue, symptom variability, and limited availability of accessible facilities as well as lack of community-based programs designed to meet their unique needs [5,6]. Seated and remotely delivered physical activity interventions may reduce some of those barriers, but success depends on aligning program content and delivery with participants’ lived experiences, preferences, and day-to-day activities.

Participant feedback is crucial to developing and refining robust interventions for individuals with chronic conditions like those with MS. The Seated Physical activity INtervention (SPIN) was developed using a three-step community-engaged research framework [6,7,8,9]. The needs and preferences for exercise training among wheelchair users with MS were first identified via one-on-one semi-structured interviews with 20 wheelchair users with MS (i.e., Step 1) [6]. The participants reported wanting aerobic and strength training that was supported by behavioral support (i.e., coaching) and could be delivered in-person or virtually. Our team identified the Guidelines for Exercise in MS (GEMS) program as an appropriate foundation for developing a wheelchair exercise training program for persons with MS [10,11,12]. The GEMS program was first adapted by experts in exercise science, behavioral science, and disability studies, then vetted by a Community Advisory Board of 10 wheelchair users with MS (i.e., Step 2) [7]. Step 3 included obtaining quantitative and qualitative feedback from individuals across 3 focus groups of 10 additional wheelchair users with MS [8]. Collectively, 40 community members contributed their knowledge and opinions to developing the SPIN exercise training program.

SPIN was recently tested in a randomized controlled trial (RCT) focused on feasibility, acceptability, and preliminary efficacy [13]. We reported evidence for feasibility and acceptability regarding SPIN [14]. This paper focally examined qualitative feedback from wheelchair users with MS who completed the SPIN exercise training program regarding program content, delivery format, barriers, and future directions for refinement. This study represents a distinct analytic step, examining participant experiences among those who completed the full SPIN intervention, and extends the broader literature on exercise training for wheelchair users beyond the primary outcomes manuscript. We believe that examining participants’ experiences related to intervention components, delivery formats, and expectations helps identify areas that can be improved to support developing robust, accessible, and meaningful programs.

## 2. Materials and Methods

This qualitative study was embedded within a RCT evaluating the SPIN intervention and a wellness comparison condition for wheelchair users with MS (NCT05888727; 5 June 2023) [13]. Participants were randomly assigned to complete the SPIN intervention condition or a wellness attention/contact control condition using REDCap randomization module created by a blinded study biostatistician. No other members of the team or participants were blinded to condition. The primary outcomes manuscript provides additional details regarding feasibility, acceptability, and safety of the study conditions. While both the exercise and wellness groups were interviewed and reported similarly high satisfaction (4.6/5) [14], this paper focuses on the exercise training condition to better understand intervention-specific mechanisms and participant experiences. Specifically, the current report is a secondary qualitative component that was designed to obtain feedback to inform the refinement of the SPIN intervention content, delivery, and future research directions.

Participants in Texas were recruited through neurology clinics, the National MS Society, the iConquer MS listserv, and MS support groups. Interested individuals completed a structured telephone screening to assess eligibility. Inclusion criteria included: (a) MS diagnosis; (b) self-reported wheelchair use, including manual wheelchair, power wheelchair, or scooter, for at least 50% of the time; (c) age 18 years or older; (d) relapse-free for the past 30 days; and (e) not currently meeting physical activity guidelines. All participants were screened for physical activity readiness, and physician consent was required for one participant. Eligible individuals completed informed consent using a REDCap form during a video conference call.

**Figure 1:**
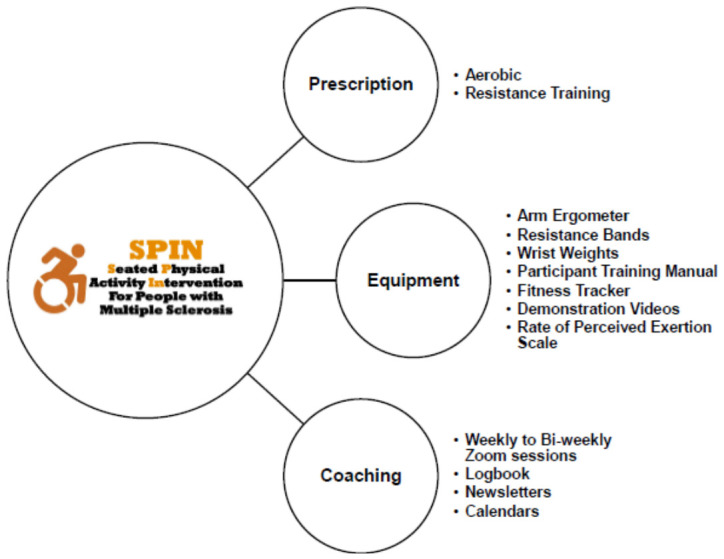
Outline of Core Components of the Seated Physical Activity INtervention for People with Multiple Sclerosis (SPIN).

SPIN is a 16-week, home-based seated exercise training program designed for wheelchair users with MS that prescribes both strength and aerobic training, which is described in greater detail in the protocol manuscript [13]. Briefly, participants were prescribed exercise sessions at least two days per week, incorporating a set of seated strength exercises using resistance bands, wrist weights, and an arm cycle ergometer for aerobic exercise (Figure 1). The targets for the exercise prescription were grounded in the Guidelines for Exercise in MS (i.e., aerobic exercise at least 2 days/week and resistance training of major muscle groups 2 days/week) [15]. Demonstration videos with modification options are provided for all resistance training exercises. Three program tracks allow participants to pursue a common exercise goal while progressing at a level aligned with their abilities and comfort. In addition to receiving the exercise prescription and equipment, participants received regular behavioral coaching sessions grounded in Social Cognitive Theory (SCT) to support adherence, self-monitoring, and behavior change [16]. The coaching sessions occur weekly for the first 8 weeks and bi-weekly for the subsequent 8 weeks, each supported by an SCT-based newsletter. Coaching sessions were led by trained behavioral coaches from the research team (SLS and AJP) with a duration of 10–30 min. Participants were provided with a comprehensive training manual, 12 newsletters, an activity monitor, a logbook, and calendars. All participants who completed the SPIN program were invited to an optional post-intervention interview following the 16-week intervention period.

Data were collected through semi-structured, one-on-one interviews conducted via video conference after completing the 16-week intervention. Video conference maximized accessibility by eliminating mobility and transportation barriers [17]. Interviews were conducted by research team members enrolled in a Master’s program, trained through graduate Qualitative Methodology coursework, and by the study Principal Investigator. Both interviewers were female and not involved in intervention delivery. The interviews followed a semi-structured interview guide developed for the study’s process evaluation aim (i.e., acceptability and opportunities for refinement). Interview domains included overall experience, enjoyable and missing components, delivery modifications, barriers, lessons learned, and additional research topics of interest. The interview guide supported consistency across interviews while allowing participants to elaborate on their experiences with the SPIN exercise training program. Participants provided verbal permission for audio recording at the time of the interview. All interviews were audio-recorded, automatically transcribed using video conference software, and reviewed for accuracy by a member of the research team prior to analysis. The transcripts were not returned to participants for comment, correction, or checking.

Data was analyzed using a rapid qualitative analysis approach [18,19], employing a deductive, domain-based analytic strategy. This approach was selected to generate timely, actionable insights to inform intervention refinement. Responses were organized according to the predefined interview questions (Table 1). Participant responses for each question were extracted and entered into a structured Excel matrix, with rows representing individual participants and columns corresponding to each interview domain. This matrix-based approach was constructed to facilitate systematic comparison across participants within each domain, consistent with rapid qualitative methods. The analytic focus was on identifying key patterns, similarities, and differences, rather than developing a hierarchical thematic structure. Two members of the research team independently reviewed the transcripts and populated the matrix, extracting and summarizing participant responses within each domain. Discrepancies in data extraction and interpretation were discussed and resolved through consensus with iterative review of the data to ensure consistency and accuracy. Patterns consistently reported across participants were prioritized, while less frequent but meaningful responses were retained when relevant to intervention refinement. To enhance transparency and rigor, the research team maintained brief analytic notes throughout the process to document emerging patterns and decision-making. Given the research team’s involvement in participant recruitment and data collection, reflexivity was addressed through ongoing team discussions to acknowledge and minimize potential bias in interpretation. These procedures were undertaken to enhance credibility, consistency, and transparency of the analytic process. Reporting was guided by the Consolidated Criteria for Reporting Qualitative Research (COREQ).

## 3. Results

**Figure 2:**
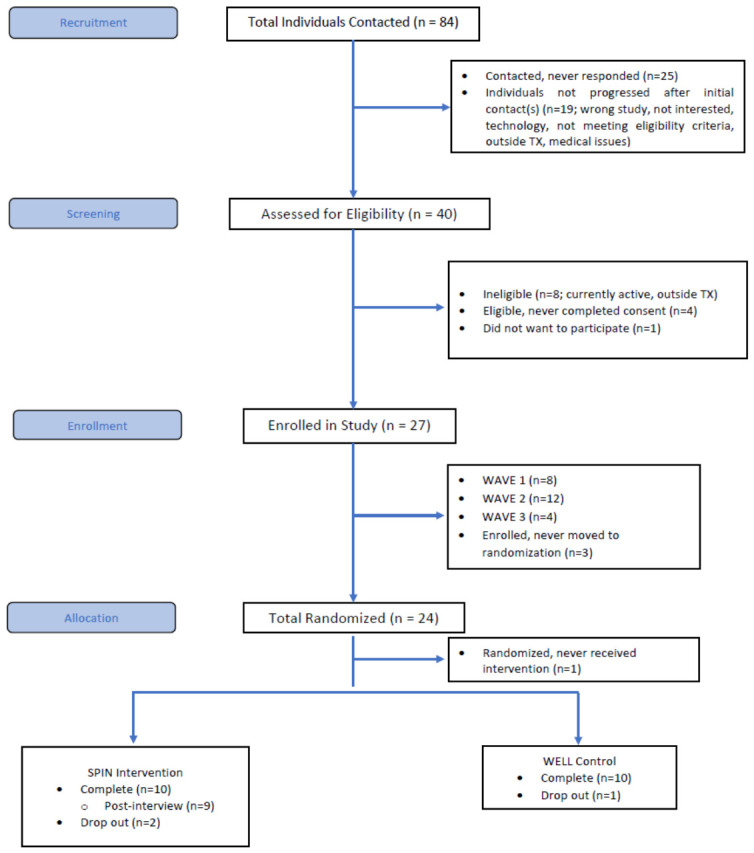
Flow Diagram Participant Enrollment and Interview Completion in the Randomized Controlled Trial Involving Wheelchair Users with Multiple Sclerosis.

Nine of the ten participants who completed the SPIN exercise training program completed a semi-structured interview (Figure 2). Demographic and clinical characteristics for the 9 participants are presented in Table 2. The mean length of interviews was 16 ± 10 min (range 7–35 min, median 13 min). Results are organized by domains: overall experience, components enjoyed, missing components, delivery modifications, barriers, lessons learned, and additional research topics of interest.

### 3.1. Overall Experience

All participants described positive experiences with the SPIN program. All reported being satisfied with their participation and emphasized they found the program enjoyable, useful, and appropriate for individuals with MS who use wheelchairs. One participant [ID008] reflected, “*It was a wonderful experience. It got me to think and move a lot better than when I was just stationary all the time… I really enjoyed it.*” Another participant [ID018] noted functional benefits, “*It definitely feels like it helps me as far as getting some strength and mobility.*”

### 3.2. Components Enjoyed

Behavioral coaching calls were a key component that six participants highlighted as offering accountability, support, and knowledge. Seven participants also highlighted that receiving exercise equipment (i.e., arm cycle and resistance bands), newsletters, and video demonstrations of resistance exercises were useful components. One participant’s quote [ID006] emphasized both the accessibility and coaching as key strengths, “*having access to do something at home was awesome, and especially working with someone that knows about neurological conditions.*”

These findings reinforce the three core SPIN intervention components—exercise prescription, equipment provision, and SCT-based coaching—as complementary mechanisms that support accessibility, self-efficacy, and sustained engagement.

### 3.3. Missing Components

Participants identified relatively few missing components within the SPIN exercise training program. Four participants provided no specific need for changes or missing components. One participant [ID019] expressed interest in live workout sessions or real-time support to improve exercise performance and ensure proper form, “*It’d be kind of beneficial to have a phone propped up or while someone’s looking at you performing that task.*” Another participant [ID023] mentioned integrating a group component or class, “*When you’re a part of a group, it makes you want to continue. Being a member of the group and not letting them down by not exercising, so that might be another thing to try.*” One participant [ID016] identified their health care provider as a key stakeholder, “*I think if the doctor understands what your program is, it would benefit not only them, but their patient-base.*”

Collectively, participants identified some missing components, with four reporting no need for changes. Suggested additions—such as live exercise sessions, group-based participation, and greater involvement of healthcare providers—highlight opportunities to enhance intervention delivery. These recommendations suggest that mechanisms related to social support, feedback, and provider endorsement may further influence engagement. Implementation strategies such as incorporating synchronous sessions, structured peer groups, and provider-facing materials or referral pathways may therefore enhance feasibility, adoption, and sustained participation.

### 3.4. Delivery Modifications

There was strong support for maintaining the SPIN program’s online format and behavioral coaching, with positive feedback on delivery from all participants. For example, one participant noted [ID004], “*I wouldn’t change the format. I like the format a lot, because meeting with [coach] was a motivator… meeting with her added accountability to it.*” At the same time, all participants suggested that adding opportunities for interaction with others could improve motivation and accountability. One participant [ID004] noted, “*I would have found it very interesting to see how others in the program were doing…some other way we could actually connect with each other in the program.*” Five participants mentioned a family member or caregiver, and two participants mentioned members of a healthcare team, such as a neurologist, physical therapist, or occupational therapist. For example, one participant [ID008] noted, “*family should be involved if they’re available, and especially your healthcare provider.*”

These findings indicate that while remote delivery and coaching support accessibility and accountability, augmenting the program with strategies to promote social support and external engagement may strengthen adherence and long-term participation.

### 3.5. Barriers

A range of barriers that affected participants’ ability to engage fully in SPIN were described. Commonly identified barriers included external factors such as scheduling constraints, equipment-related issues, and limited space within the home environment. One participant [ID006] mentioned the initial equipment set-up, “*The only problem I had was with the equipment, I was lucky cause my son was here putting it together. That would have been a challenge if he wasn’t.*” Three participants described difficulty maintaining consistency over time when experiencing fatigue, competing priorities, or a period of inactivity. One participant [ID004] mentioned that “*The most challenging part is starting… I went like maybe a week and a half without doing anything. It was like starting all the way over from scratch.*” Three participants noted functional challenges. For example [ID016], “*Getting to a position of being able to use the machine or the rubber bands. Being limited in mobility in a wheelchair, it takes an effort sometimes for me to get there.*”

These findings suggest that engagement is influenced by multiple interacting mechanisms, including physical capability, environmental context, and self-regulation over time. Implementation strategies such as enhanced onboarding and equipment support, flexible and adaptable delivery formats, and targeted approaches to support re-engagement after lapses may help mitigate these barriers. Additional tailoring of the SPIN intervention to accommodate functional limitations may further strengthen accessibility and sustainability.

### 3.6. Lessons Learned

Participants reported learning meaningful lessons through their participation such as increased self-awareness related to exercise training and health behaviors, along with greater motivation and accountability. One participant [ID016] reflected that the program encouraged them to “*Take advantage of every moment that you have and to try to get better.*” Four participants emphasized the importance of consistency, routine, and self-discipline in maintaining exercise while facing barriers [ID018], “*Just trying to motivate myself to continue with the program, knowing that it is beneficial.*” Four participants noted that these lessons extended beyond the intervention period and applied to functioning in daily life [ID004], “*Now I’m doing 3 and 4 days a week. I want this to be a regular part of my routine, because I know if I go a few days without doing anything, then I can feel this spasticity getting worse.*” This comment suggests their experience participating in SPIN may support long-term behavior change and self-management.

Participants reported increased self-awareness, motivation, and accountability, emphasizing the role of consistency and routine in sustaining exercise behaviors. These lessons extended beyond the intervention period, suggesting potential for longer-term behavior change. These findings highlight the importance of mechanisms such as self-regulation and habit formation. From an implementation standpoint, incorporating strategies to support maintenance, including goal setting and self-monitoring, may help sustain engagement over time.

### 3.7. Additional Research Topics of Interest

Four participants expressed interest in future research addressing MS-specific wellness topics that could complement the SPIN intervention. Common wellness topics mentioned were diet and fatigue, which participants described as relevant to daily functioning and overall well-being. For example [ID004], “*Something with diet… with what you’re eating and drinking would be super beneficial.*” Two participants mentioned advances in MS research, specifically medications and assistive technology [ID016], “*education on the different medicines that doctors are prescribing or offering education on other studies.*”

These findings suggest that engagement may be influenced by perceived relevance to broader health goals. Implementation strategies that integrate complementary wellness content or connect participants to additional resources may therefore enhance intervention engagement, acceptability, and sustainability.

Table 3 summarizes, by domain, key findings and implications for SPIN intervention refinement, with representative quotes.

## 4. Discussion

This qualitative study examined formative feedback from wheelchair users with MS who participated in the initial testing of the SPIN exercise training program. The findings provide insight into the program’s perceived strengths, areas to refine, and potential strategies to optimize future iterations of exercise-based interventions for this population. Participants reported positive overall experiences in SPIN and identified opportunities to enhance the program. Results highlight potential behavioral and implementation mechanisms through which the SPIN exercise training program may influence behavior and outcomes.

Across interview domains, participants emphasized the importance of clarity and guidance within the intervention. Although the program was generally well-received, one participant expressed a desire for additional real-time support to increase confidence during exercise sessions and ensure proper form. This notion hints at the importance of self-efficacy and feedback within SCT for engaging in independent exercise. More broadly, across domains, the participant responses map to key SCT constructs including self-efficacy, social support, outcome expectations, and self-regulation, which collectively drive sustaining physical activity behavior [16,20].

Participants shared their preferences about program delivery. Participants consistently noted that the online format was a strength because it was convenient and accessible for individuals who manage mobility limitations and symptom variability. The SPIN program was purposefully designed as an online intervention to address key limitations of prior programs for wheelchair users with MS, including home-based manual wheelchair propulsion and center-based Pilates [21,22], which do not sufficiently accommodate heterogeneous functional needs or overcome transportation barriers. Five participants also expressed interest in having greater opportunities to interact with other program members or other knowledgeable professionals. This idea is consistent with previous research that reported social support and peer interaction can enhance motivation and adherence in MS and other populations [23,24,25]. Future iterations of the SPIN exercise training program may benefit from maintaining the flexibility of remote delivery while incorporating optional opportunities for peer interaction to enhance motivation and accountability. Such adaptations may strengthen social support and accountability mechanisms; however, they also require consideration of implementation trade-offs, including scheduling logistics, participant privacy, and costs related to staffing demands that can limit scalability.

Participant reported barriers and challenges were largely contextual rather than program-specific. Participants identified scheduling constraints, weather conditions, equipment set-up, and limited space as challenges that affected participation. These barriers reflected broader realities of living with MS as a wheelchair user rather than limitations of the SPIN program. Notably, participants also identified facilitators that supported their engagement, including the program’s structured format, clear instructions, and the ability to complete exercises at home. Together, these highlight the importance of a balanced, flexible structure when designing interventions for this population. The pattern is also consistent with prior research in MS, where fatigue, environmental constraints, and competing demands frequently influence exercise participation [26,27,28].

Participants described learning meaningful lessons throughout the SPIN exercise training program. Previous literature indicates that key behavior change techniques used in MS exercise training programs include instructions on how to perform the behavior, social support, demonstration of the behavior, credible source, self-monitoring of behavior, and graded tasks [29]. These components are all directly integrated within the SPIN exercise training program curriculum, but were also noted as key facilitators by our participants. Specific SPIN components they described as useful were the resistance training demonstration videos and social support offered by the behavior coach, who provided supportive accountability. Additionally, participants described having overall positive experiences with the SPIN program and specifically highlighted the program’s accessibility, structure, and adaptability as key strengths. The collective findings from this qualitative analysis offer participant-informed direction to further refine the SPIN intervention and support its continued development as an accessible, participant-centered exercise program.

Future research should extend beyond feasibility and participant experience to evaluate health outcomes associated with exercise training among wheelchair users with MS [30,31]. There is strong evidence for the benefits of exercise among persons with mild-moderate MS, including physical function, cognition, and fatigue [3,4,32]. Accordingly, future iterations of SPIN could incorporate outcomes related to physical and cognitive performance, fatigue, and neural function to better understand the full impact of the intervention and its potential role in supporting health among wheelchair users with MS.

Several study limitations should be considered when interpreting the findings. The sample size was small and limited to participants who completed the SPIN intervention condition. This may limit generalizability among the broader population of wheelchair users with MS. The qualitative data were collected after the intervention and relied on participant recall; the shortest interview lasted 7 min, potentially limiting the data’s richness. Responses may have been influenced by social desirability, as interviews were conducted by research team members who had previously screened and consented the participant, however participants were never interviewed by their behavioral coach. Although findings were consistently positive, they should be interpreted in the context of study limitations, including interviewer non-independence, reliance on post hoc recall, and potential selection bias from including only participants who completed the intervention. The rapid qualitative analysis approach may have limited the depth of analysis as compared to using alternative qualitative methods. Yet, this approach was intentionally selected to generate timely, actionable insights to inform refinement of the intervention. Despite these limitations, the study provides valuable, participant-informed insights into the design and delivery of a home-based exercise intervention for wheelchair users with MS. The findings offer practical direction for refining the SPIN program and support ongoing efforts to develop an accessible, scalable, and behaviorally grounded exercise training program for this population.

## 5. Conclusions

Findings from this formative qualitative analysis offer insights into how the SPIN exercise training program can be refined to better meet the needs of wheelchair users with MS. Participant feedback highlights the importance of flexibility and social support in fostering engagement and promoting sustained behavior change. These findings will inform future iterations of the SPIN program and contribute to research focused on accessible participant-centered lifestyle interventions for individuals with disabilities.

## Figures and Tables

**Table 1:** One-on-one Semi-structured Interview Guide for Formative Feedback on the SPIN Exercise Training Program.

Question	Domain
Can you describe your overall experience in the SPIN program?	Overall Experience
What are some aspects of the program that you enjoyed?	Components Enjoyed
What are some aspects you think are missing or would help improve the program?	Missing Components
The SPIN program was delivered online, one-on-one with a behavioral coach. Can you please describe any ways you would change this format?	Delivery Modifications
What was the most challenging part of completing the study for you?	Barriers
What did you learn from participating in the study that will be helpful as you move forward?	Lessons Learned
What other research topics would you be most interested in participating in?	Additional Research Topics of Interest

**Table 2:** Demographic and Clinical Characteristics of Nine Participants who Completed Interviews Following the SPIN Exercise Training Program.

Variable, *Units*	
**Age**, *years (SD)*	56.6 (10.2)
**Disease Duration**, *years (SD)*	10.3 (9.4)
**Patient Determined Disease Steps**, *median (IQR)*	7 (0)
**Wheelchair Type**, *n (%)*	
Power Wheelchair	4 (44)
Manual Wheelchair	3 (33)
Power Scooter	2 (22)
**Biological Sex**, *n (%)*	
Female	6 (67)
Male	3 (33)
**Race**, *n (%)*	
Caucasian	5 (56)
African American/Black	4 (44)
**Education**, *n (%)*	
High School or Some College	3 (33)
College Degree or More	6 (67)
**Marital Status**, *n (%)*	
Married	5 (56)
Single/Divorced/Separated/Widowed	4 (44)
**Employment Status**, *n (%)*	
Yes	2 (22)
No	7 (78)

**Table 3:** Summary of Qualitative Findings and Implications for SPIN Intervention Refinement.

Domain	Key Findings	Representative Quote	Implications for Intervention Refinement
Overall Experience	Participants reported high satisfaction and perceived functional benefits.	“It was a wonderful experience… got me to think and move a lot better.” [ID008]	Maintain core program structure and quantify functional outcomes in future trials.
Components Enjoyed	Coaching, equipment, and educational materials supported accountability and accessibility.	“Having access… and working with someone that knows neurological conditions.” [ID006]	Preserve multimodal delivery; reinforce coaching as a key engagement strategy.
Missing Components	Interest in live sessions, group interaction, and healthcare provider involvement.	“When you’re part of a group, it makes you want to continue.” [ID023]	Incorporate synchronous sessions, peer groups, and provider engagement strategies.
Delivery Modifications	Strong support for the online format, with a desire to increase involvement from family, peers, and healthcare providers.	“Family should be involved if they’re available, and especially your healthcare provider.” [ID008]	Maintain remote delivery while integrating opportunities for social connection.
Barriers	Scheduling, equipment setup, fatigue, and functional limitations impacted engagement.	“The most challenging part is starting…” [ID004]	Enhance onboarding, flexibility, and re-engagement supports; tailor to functional needs.
Lessons Learned	Increased self-awareness, routine, and sustained behavior change.	“Just trying to motivate myself to continue with the program, knowing that it is beneficial.” [ID018]	Strengthen self-monitoring, goal setting, and maintenance strategies.
Additional Topics	Interest in diet, fatigue, and MS-related education.	“Something with diet…with what you’re eating and drinking would be super beneficial.” [ID004]	Integrate complementary wellness content and resource linkage.

## Data Availability

The datasets presented in this article are not readily available because the qualitative nature of the data is a concern for confidentiality.

## Abbreviations

The following abbreviations are used in this manuscript:

MS	Multiple Sclerosis
SPIN	Seated Physical Activity INtervention
GEMS	Guidelines for Exercise in MS
RCT	Randomized Controlled Trial
SCT	Social Cognitive Theory
